# Mechanism mapping to advance research on implementation strategies

**DOI:** 10.1371/journal.pmed.1003918

**Published:** 2022-02-08

**Authors:** Elvin H. Geng, Ana A. Baumann, Byron J. Powell

**Affiliations:** 1 Center for Dissemination and Implementation, Institute for Public Health and Division of Infectious Diseases, Department of Medicine, Washington University School of Medicine, St. Louis, Missouri, United States of America; 2 Division of Prevention Sciences, Department of Surgery, Washington University School of Medicine, St. Louis, Missouri, United States of America; 3 Center for Dissemination and Implementation, Institute for Public Health and Brown School, Washington University, St. Louis, Missouri, United States of America

## Abstract

Elvin Hsing Geng and colleagues discuss mechanism mapping and its utility in conceptualizing and understanding how implementation strategies produce desired effects.

To advance as a science, implementation research must build an evidence base using, in part, experimental designs. But trials of implementation strategies—which are often complex health systems interventions—often do not demonstrate anticipated overall effects [1,2]. As a consequence, studies to understand whether a strategy will work are strongest when they can also reveal how they succeed or fail. The trial by Sarkies and colleagues published recently in *PLOS Medicine* illustrates this need [3].

Previous randomized trials and a meta-analysis provided evidence that weekend staffing with allied healthcare professionals (e.g., physical therapists) decreased length of stay in rehabilitation units, but such weekend staffing had no effects in general medical and surgical units [4]. Because staffing in real-world practice does not reflect these data [5], Sarkies cluster-randomized 45 healthcare units to receive one of 3 conditions: usual practice (the control); written materials about staffing evidence; or a knowledge broker (KB)—defined in this study as “intermediary agents who build relationships between decision makers and researchers, by sharing expert knowledge and establishing communication channels to convey evidence.” The study found that neither written dissemination nor a KB had an effect on staffing nor patient length of stay compared to usual practice. Why? Data about implementation showed that managers assigned to a KB had very limited actual interaction with the KB and attended very few meetings. But additional questions remain. Why was KB attendance poor? Was the knowledge itself or the broker of the knowledge unconvincing? Which contextual enablers were missing? The answers to these questions are crucial for understanding the prospects of KB as a useful implementation strategy. Understanding how strategies fail is no less important than identifying success.

One way to dissect how implementation strategies fail is through formal exploration of anticipated mechanisms. Several approaches in implementation science offer methods that can help conceptualize mechanisms systematically. The Intervention Mapping approach asks those planning health promotion activities to conceive of the multilevel, theory-based targets for behavior change that underlie a program and how they interact to produce desired effects [6]. The Theory of Change approach encourages programs to make their intentions, activities, steps behind change, and assumptions explicit through a visual diagram [7], drawing from literature review, contextual knowledge, and stakeholder-engaged methods. “Mechanism mapping” (a concept from policy analysis) [8] advocates decomposing an effect of interest into its component steps and asking how those steps interact with context. Directed acyclic graphs which have become widely used in epidemiology can be conceived of a tool for representing how an effect occurs mechanistically. A mechanistic exploration of the KB strategy tested by Sarkies and colleagues reveals potential routes of effect, accompanying assumptions and contextual influences, and thereby points us toward several key scientific opportunities.

First, decomposing the overall effects of an implementation strategy—in this case KB—into its hypothesized mechanistic component allows investigators to be more precise about where it may have broken down. Drawing from previous literature [9], we suggest KB acts through 3 potential pathways (Fig 1): forming social links with the target managers (step a); offering curated “evidence” of staffing to managers (step b); and conveying skills to enable use of staffing evidence (step c). In turn, these influence the manager’s capability, opportunity, and motivation (steps e and f) to use an evidence-based intervention (step g). Such a mapping conceptualizes KB as a multipathway approach that acts through affective (forming a bond), cognitive (brokered knowledge), social (linking to a network), and instrumental (capacity building) means. Visual mapping also reveals temporal dependencies. For example, forming a social bond (step a) likely needs to come first to magnify subsequent effects of conveying knowledge of evidence (step b). Mapping also makes explicit the need to draw from social network theory, facilitation, organizational theory, and diffusion of innovation among others [10,11]. In fact, such a decomposition can be seen as a “theory of the strategy” that helps researchers interrogate, revise, and build toward a general, scientific understanding of KB. In this case, mapping identifies several points of potential weakness in this version of the KB strategy: limited intention to first cultivate a relationship between KB and managers (step a), possible attenuated rapport formation via web-based connection, and less attention on skills development (step d).

**Fig 1:**
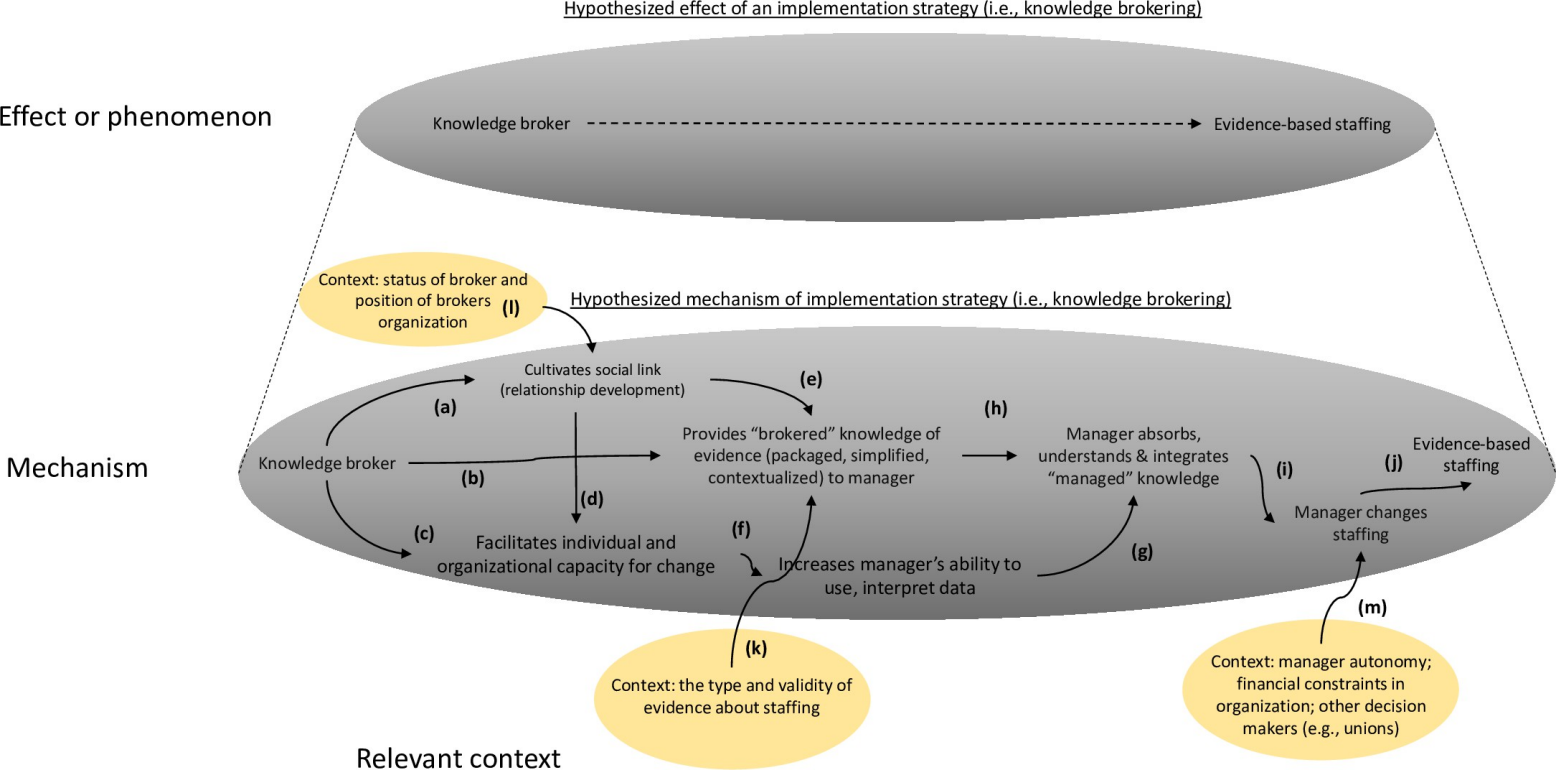
The mechanism of the effect of an implementation strategy can be conceived of as being composed of a chain of events (in the larger ellipse below) underlying a larger phenomenon (smaller ellipse above) [18]. In this case, we synthesize and simplify existing literature to suggest that knowledge brokering potentially occurs through (a) developing a relationship with the knowledge recipient; (b) offering evidence to the recipient; and (c) building capabilities to use that evidence in the recipient. These activities, in turn, are hypothesized to increase the capability, motivation, and opportunity of the manager to apply evidence-based intervention, which may then lead to changes in behavior (step i). Contextual effects are represented as ellipses pointing into specific nodes in the chain of event (steps k, l, and m). We use a convention from some causal diagrams in which 2 arrows pointing into 1 node (i.e., word) implies effect modification. *Image credit*: *Raymond Craver*.

A visual mapping of the putative mechanism also highlights underlying assumptions and their uncertainties and therefore informs what to measure. The effect of conveying evidence (step b) assumed the evidence is perceived as credible. A mixed-methods manuscript [12] from the same study (which should be considered best practice) investigates these assumptions and finds it wanting: Managers in the targeted hospitals did not accept the “evidence” about staffing because in part they felt the “average effects” in trials were not applicable to their specific settings. Skepticism about the evidence likely explained the observed limited participation by managers in KB webinars after initial contact. Another assumption is that the manager would accept knowledge from the particular broker in this strategy (a postdoctoral scholar) who may not be of high status in their eyes (steps a and b) [13]. The study reveals little about whether this assumption held. Finally, mapping demonstrates the assumption that the manager (the action target) has sufficient authority to make changes (step i)—another unknown [14]. Additional details confirming or contesting these assumptions would further help explain lack of effects but were not present in the study.

Mechanism mapping also increases the rigor of a study through making explicit where and when particular elements of the context act on particular steps in the hypothesized chain of events. Several elements of context potentially differ between the study by Sarkies and colleagues and other settings using KB, differences that could inform external validity of null effects if better measured. For example, in this case, the nature of the evidence offers one important contextual element: It could be that evidence of a managerial practice in this study is perceived as fundamentally different from evidence of a clinical treatment and thereby attenuated the effect of brokered knowledge on target manager beliefs (step k). Additionally, the organizational relationship between the KB agency and the hospital may have a critical influence on the success of the social and affective link between the KB and the manager (step l) [15]. Other potential contextual factors exist (step m). Decomposing a hypothetical effect enables us to conceive of context with greater precision by naming its influences more precisely [16].

Sarkies and colleagues’ new paper moves implementation science ahead in 2 ways. First, it suggests that KB depends on the status of the actor, the means of contact, the perceived applicability of evidence, the affective bond created by the KB, and the interactions between each of these factors. Future studies of KB must emphasize these elements. Second, and more broadly, the study reminds us that trials of complex health systems interventions must allow us to explain whether a strategy was used and, if not, why not [1,2,17]. In this study, a rigorous conceptualization of the strategy and clear description of actor, action, and other components allowed us to see the that poor engagement with the KB explained null effects and also that credibility of the evidence to managers was in part to blame. Systematic mechanism mapping could potentially help yield additional insights. A failed intervention may yet be a successful study when it provides generalizable insights. Explicitly specifying and measuring hypothesized mechanisms of implementation strategies can help us make the most of our investments in implementation research.
